# 110 Cases of Syphilis Treated by the Ehrlich-Hata Method

**Published:** 1911-03

**Authors:** 


### 110 Cases of Syphilis Treated by the Ehrlich-Hata Method

MICHAELIS (*Berliner klinische Wochenschrift,* Jahrgang 47, No. 37) reports in detail the treatment of 110 cases of syphilis with the Ehrlich-Hata preparation. In seven cases treated for the primary sore, the lesion disappeared in one to three weeks without any local treatment. One of these cases had already reached the stage of spontaneous disappearance, and in it twenty-four hours after the injection of 0.6 of a gramme of the remedy there appeared a rather circumscribed maculopapular eruption, which, however, had disappeared within another twenty-four hours. In the second group were 15 cases of secondary syphilis, previously untreated, showing skin exanthemata of different kinds, specific sore throat, and mucous patches. These cases all reacted exceptionally well, although the time required depended upon the difference in the anatomical character of the lesion. As a rule four to twelve days were required for the complete disappearance of the eruption. One case of acne eruption of the scalp, which was certainly specific in nature, was very resistant.

There were treated 22 cases of secondary syphilis which had recurred after mercurial treatment. In this group were four cases of severe brain syphilis, with papuloedema, three of which were very favorably influenced; the fourth had not been under observation long enough at the time of making the report to determine the result.

The most wonderful results were shown in 33 cases, with very severe syphilitic symptoms, which in spite of repeated and often long-continued treatment with mercury and iodides had not been freed of their symptoms. Amongst these were a number of cases which would have come to a fatal end in a short time, others who had been unable to work for years, others with persistent ozena which had rendered them loathsome to associates, patients with wide-spread ulcerating gummata of the skin, nose, lips, and tongue, and those with severe nocturnal bone pains. Such cases were relieved in a few days after the administration of the remedy, although they had been afflicted for months or years and had failed to yield to the most persistent treatment. The only cases which remained uninfluenced were a few which suffered from old spinal and cerebral syphilis, and in which there was doubtless extensive degeneration of the nerve substance.

Of congenital syphilis 10 cases were treated, four being in sucklings and six of late manifestation. Of the sucklings, two were critically ill with severe visceral lues and died twenty-four hours after injection.

It is difficult to say whether or not the treatment hastened death, because the children were almost in a state of collapse when the remedy was administered. A third case suffering from severe skin lesions was free from symptoms six days after injection.

There were also treated 15 cases of tabes and 8 of paralysis. In far advanced cases of this sort this preparation, as is to be expected, is of no use. In incipient cases the results are favorable, and injection in such cases is without harm. In several cases of tabes which were in the initial stage, although they showed marked ataxia, the ataxia became much less after the injection. In one case of paresis there was a marked remission, but as this at times occurs spontaneously, it cannot with certainty be attributed to the injection.—*Therapeutic Gazette.*

